# Subtle radiographic findings of pneumomediastinum triggered by singing

**DOI:** 10.1093/omcr/omag135

**Published:** 2026-07-27

**Authors:** Tatsuhiro Amano, Atsuo Maeda, Hikaru Kawakita, Tomoko Yashima, Go Haraguchi, Masahito Uchino

**Affiliations:** Department of Emergency and Critical Care Medicine, Kawakita General Hospital, 1-6-1 Asagaya-kita, Suginami-ku, Tokyo 166-8588, Japan; Department of Emergency and Critical Care Medicine, Kawakita General Hospital, 1-6-1 Asagaya-kita, Suginami-ku, Tokyo 166-8588, Japan; Department of Emergency and Critical Care Medicine, Kawakita General Hospital, 1-6-1 Asagaya-kita, Suginami-ku, Tokyo 166-8588, Japan; Department of Emergency and Critical Care Medicine, Kawakita General Hospital, 1-6-1 Asagaya-kita, Suginami-ku, Tokyo 166-8588, Japan; Department of Emergency and Critical Care Medicine, Kawakita General Hospital, 1-6-1 Asagaya-kita, Suginami-ku, Tokyo 166-8588, Japan; Department of Emergency and Critical Care Medicine, Kawakita General Hospital, 1-6-1 Asagaya-kita, Suginami-ku, Tokyo 166-8588, Japan

**Keywords:** Pneumomediastinum, continuous diaphragm sign, Naclerio’s V sign, asthma, chest radiography, computed tomography

**Figure 1:**
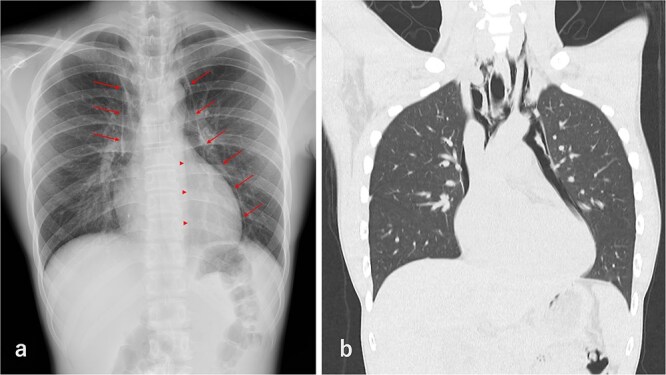
(a) Gas collection between the visceral and parietal pleura (arrows) and a lucent area along the left border of the descending aorta (arrow heads). (b) Sagittal plain chest computed tomography image.

A 15-year-old Japanese male, tall and lean, with a history of bronchial asthma was admitted to our hospital. He complained of persistent anterior chest pain following a choir class. The only subjective symptom was anterior chest pain that worsened during inspiration. He denied cough, dyspnea, or vomiting. Vital signs were stable, with no abnormal breath sounds or chest wall findings. Chest radiography demonstrated a contour line extending from the main pulmonary artery to the left ventricle of the heart, representing the visceral and parietal pleura separated by gas. Additionally, a lucency parallel to the left side of the descending aorta, alongside subcutaneous emphysema in the neck were noted. No pneumothorax was observed (Fig. 1a). Chest computed tomography confirmed mediastinal emphysema extending from the pericardium to the neck (Fig. 1b). The patient was diagnosed with idiopathic pneumomediastinum, likely secondary to an acute intrathoracic pressure increase during singing. He was managed conservatively. Follow-up radiography on day 10 showed near-complete resolution.

Pneumomediastinum is a rare condition, featuring leakage of air into the mediastinum. Asthma-related air trapping predisposes to alveolar rupture during forceful expiration, with air tracking along bronchovascular sheaths into the mediastinum (Macklin effect) [1]. Thus, asthma itself may serve as a predisposing condition for pneumomediastinum, regardless of acute exacerbation [2]. In our patient, the underlying asthma likely provided this predisposition, while singing—causing a sudden rise in intrathoracic pressure—acted as the precipitating trigger. While the continuous diaphragm and Naclerio's V signs are hallmark radiographic findings of pneumomediastinum, they may not be clearly evident, as demonstrated in the current case. In conclusion, when mediastinal emphysema is clinically suspected, clinicians should carefully evaluate chest imaging for subtle findings indicative of the condition, including linear lucencies outlining the mediastinum and great vessels, rather than relying solely on the presence of classic radiographic signs.
